# Identification of Incident Uterine Fibroids Using Electronic Medical Record Data

**DOI:** 10.5334/egems.264

**Published:** 2019-03-29

**Authors:** Onchee Yu, Susan D. Reed, Renate Schulze-Rath, Jane Grafton, Kelly Hansen, Delia Scholes

**Affiliations:** 1 Kaiser Permanente Washington, US; 2 University of Washington, US; 3 Bayer Pharma AG, DE

**Keywords:** algorithms, classification and regression tree, electronic health records, diagnosis codes, positive predictive value, uterine fibroids, uterine leiomyomata

## Abstract

**Introduction:**

Uterine fibroids are the most common benign tumors of the uterus and are associated with considerable morbidity. Diagnosis codes have been used to identify fibroid cases, but their accuracy, especially for incident cases, is uncertain.

**Methods:**

We performed medical record review on a random sample of 617 women who received a fibroid diagnosis during 2012–2014 to assess diagnostic accuracy for incident fibroids. We developed 2 algorithms aimed at improving incident case-finding using classification and regression tree analysis that incorporated additional electronic health care data on demographics, symptoms, treatment, imaging, health care utilization, comorbidities and medication. Algorithm performance was assessed using medical record as gold standard.

**Results:**

Medical record review confirmed 482 fibroid cases as incident, resulting a 78 percent positive predictive value (PPV) for incident cases based on diagnosis codes alone. Incorporating additional electronic data, the first algorithm classified 395 women with a pelvic ultrasound on diagnosis date but none before as incident cases. Of these, 344 were correctly classified, yielding an 87 percent PPV, 71 percent sensitivity, and 62 percent specificity. A second algorithm built on the first algorithm and further classified women based on a fibroid diagnosis code of 218.9 in 2 years after incident diagnosis and lower body mass index; yielded 93 percent PPV, 53 percent sensitivity, and 85 percent specificity.

**Conclusions:**

Compared to diagnosis codes alone, our algorithms using fibroid diagnosis codes and additional electronic data improved identification of incident cases with higher PPV, and high sensitivity or specificity to meet different aims of future studies seeking to identify incident fibroids from electronic data.

## Introduction

Uterine leiomyomata, or uterine fibroids, are common benign tumors of the uterus that are associated with considerable health care costs [1] and morbidity including excessive bleeding, pelvic pain, urinary symptoms, poor pregnancy outcomes, infertility, and other adverse health outcomes [2]. In the United States, the cumulative incidence of uterine fibroids by age 50 has been estimated to be over 80 percent for black women and nearly 70 percent for white women [3]. Despite the public health burden, uterine fibroid treatment options remain limited. Hysterectomy is the definitive cure for symptomatic women [4]. Other therapeutic interventions include myomectomy for women who wish to preserve future fertility, which can temporarily improve symptoms and reproductive outcomes [4]. Pharmacologically, gonadotropin-releasing hormone agonists, selective progesterone modulator and levonorgestrel intrauterine system are currently the only agents approved for medical management of fibroid symptoms. However, these agents can have side effects, and relapses follow closely on discontinuation [5]. Thus, additional therapeutic options are needed.

In order to assess other medical therapies for fibroids and to allocate resources appropriately, it is important to have methods that can accurately identify large samples of women with fibroid. The widespread availability of detailed electronic health record (EHR) data provides the opportunity to identify such samples with great economy. Diagnosis codes in the EHR have been used to identify symptomatic uterine fibroid cases [1,6,7,8]. However, their accuracy, especially for identifying incident cases, is understudied and uncertain. In view of this gap, we undertook an evaluation of the accuracy of the diagnosis codes available from a health plan EHR to identify incident uterine fibroid cases. We then used additional potential predictors collected from the same automated databases to develop classification algorithms for incident fibroids, with the goal of improving case identification. We focused on either higher sensitivity (i.e., maximizing the inclusion of true incident fibroid cases) or higher specificity (i.e., avoiding incorrectly including non-cases as incident fibroid cases). We focused on algorithms that would identify incident cases, as opposed to prevalent cases, since we were most interested in defining a population of newly diagnosed women, rather than a group of women who might have had uterine fibroids for a number of years and perhaps have tried and failed an intervention. The accurate identification of women with incident uterine fibroids could assist researchers in targeting interventions for newly diagnosed symptomatic women with uterine fibroids.

## Methods

### Study setting

The parent cohort study collected data from January 2005 through December 2014 at Kaiser Permanente Washington (KPWA), an integrated health care delivery system in the United States that provides comprehensive health care and insurance coverage to about 600,000 members in Washington state and parts of Idaho. KPWA contracts with the Kaiser Permanente physician group to provide care within an integrated group practice division for approximately 70 percent of the plan’s enrollees. The remaining 30 percent receive care from non-KPWA contracted provider networks located in areas not served by KPWA medical centers. KPWA maintains information on demographics, health plan enrollment, health care utilization, inpatient and outpatient diagnoses and procedures, pharmacy dispensing, radiology and laboratory tests and results, vital signs, and death in their automated databases. All encounters at KPWA-owned clinics as well as claims from outside facilities are included, and all data are linked by a unique consumer number assigned to each enrollee. Beginning in 2005, all patient care contacts have been recorded in a fully integrated EHR system at all KPWA-owned clinics.

KPWA is one of 18 participating sites within the Health Care Systems Research Network (HCSRN). The Virtual Data Warehouse (VDW) that includes all of the data elements described above was the primary source of data in our study [9]. The VDW is a common data model that is shared by health systems within the HCSRN. It is a set of mutually agreed upon data standards and processes that allow data extraction programs written at one implementing site to be run quickly and efficiently against VDW at other sites while minimizing site-specific customization. All of our study data that were used to identify potential fibroid cases, and potential predictors were obtained from the KPWA’s VDW. The study was approved by the KPWA Institutional Review Board.

### Selection of potential incident uterine fibroid cases

Women were included as potential incident uterine fibroid cases if they met all of the following selection criteria:

1. aged 18–65 years during 2012–2014,
2. resided in Western Washington and received care in the group practice division,
3. had an International Classification of Diseases, 9^th^ revision, Clinical Modification (ICD-9) uterine fibroid diagnosis code (218, 218.0, 218.1, 218.2, 218.9) as part of an in-person medical encounter (inpatient, outpatient, and/or emergency department) during 2012–2014,
4. were continuously enrolled at KPWA for at least 3 years prior, and
5. had at least 1 health care utilization (medical encounter, pharmacy fill, laboratory work, etc.) at KPWA in the past 3 years.

Women who had a history of hysterectomy as identified from the procedure codes in the automated data were excluded. Women who had a fibroid diagnosis code in the past 3 years were also excluded as indicative of prevalent cases. A total of 2,351 women met the selection criteria during the 3-year study period and a random sample of 617 women were selected for medical record review.

To explore if there were any true incident uterine fibroid cases that did not have a fibroid diagnosis code, we also selected a random sample of 70 women from 5,355 who met all of the above selection criteria but who, while not having a fibroid diagnosis during the study period, had procedures and/or symptom codes that were common for fibroids recorded in the automated databases: uterus preserving procedures (myomectomy and ablation), hysterectomy, and/or heavy uterine bleeding. In a previous UK study, these procedures and conditions were found to include some confirmed fibroid cases [10].

### Data collection

A trained abstractor reviewed the medical records to ascertain the true incident case status for the selected random samples. The abstractor was instructed to collect information from radiology reports (pelvic and obstetrical ultrasound, hysterosalpingogram, saline infused sonohysterogram, pelvic CT, and MRI), uterine pathology findings, and medical encounters, all of which were available in the EHR. To confirm case status, the abstractor looked for evidence of uterine fibroid in these reports, allowing a window of 60 days before and 60 days following the fibroid ICD-9 diagnosis date. To determine if a confirmed case was an incident case, history of fibroid documented in the EHR any time prior to the 60-day window leading up to diagnosis was collected. In instances when the abstractor was uncertain of case status, the study clinician (S.D.R) reviewed the EHR and determined the final status. Abstractor and study clinician were blinded as to the potential case group from which a woman had been selected (with or without a fibroid diagnosis code).

We collected potential predictors of incident uterine fibroids from the automated data for the classification algorithm development. Table 1 shows the data elements and the corresponding tables in the VDW where the data were stored. Specifically, we collected fibroid visit setting (inpatient, outpatient, and emergency department) as well as demographics including race and ethnicity, body mass index (BMI), and smoking status. Menopausal status and education were collected but were not included as potential predictors in the models due to a large amount of missing (47 percent and 80.7 percent, respectively). Other variables collected during the 3 years prior through 2 years after the fibroid diagnosis included potential fibroid symptoms, treatment, radiological imaging, health care utilizations, other comorbidities and medication use (Table 1).

**Table 1:** Potential predictors of incident uterine fibroid evaluated in the classification algorithm development.

Category	Variables	Virtual Data Warehouse Table

Demographics	Age, race, ethnicity, body mass index, smoking status	Demographics
Fibroid Symptoms	Heavy uterine bleeding, other bleeding symptoms, urinary incontinence, abdominal pain, pelvic pain	Inpatient and outpatient diagnosis codes from utilization table
Imaging	Pelvic ultrasound, pelvic CT, pelvic MRI, hysterography	Procedure codes from utilization table
Health Care Utilization	Primary care visit, hospitalization, OB/GYN visit, all utilizations including telephone encounters and secure messaging	Medical encounters in utilization table
Comorbidities	Charlson comorbidity index, breast cancer, atypical ductal hyperplasia, ovarian cancer, fallopian cancer, endometrial cancer, endometrial hyperplasia, uterine cancer, colon cancer, lung cancer, endometriosis, adenomyosis, diabetes, depression	Inpatient and outpatient diagnosis codes from utilization table
Medication use	Combined oral contraceptives, levonorgestrel intrauterine system, progesterone, estrogen, fertility, steroids, GnRH analog, androgen, aromatase inhibitor, mifepristone, NSAIDs, opioids, tranexamic acid, clomifene, raloxifene, tamoxifen, toremifene, ospemifene, bazedoxifene	Medication fills from pharmacy table

### Definition for a true incident uterine fibroid

A true incident uterine fibroid was defined as having evidence of a fibroid documented in radiology reports, pathology findings and/or medical encounters during the 60 days before and after the ICD-9 diagnosis date, with no fibroid present and no history of fibroid noted prior to this time window, as determined by the medical record abstraction.

#### Statistical Analyses

To assess the accuracy of the ICD-9 diagnosis codes in identifying incident uterine fibroids, we calculated positive predictive values (PPV), defined as the percentage of women with an ICD-9 diagnosis code for fibroids in the automated database who were confirmed with an incident fibroid from medical record review. The 95 percent confidence intervals (CI) for PPVs, based on the exact binomial distribution, also were calculated.

Using additional potential predictors identified from the automated data, we conducted a classification and regression tree (CART) analysis to develop classification algorithms for incident uterine fibroids [11]. CART is a binary recursive partitioning method that builds a decision tree that classifies individuals as having or not having a true event (in this case, a true incident uterine fibroid) based on a set of predictors. At each partitioning step, the model evaluates all possible binary splits of all potential predictors and partitions the sample into 2 subgroups at the optimal split that yields the best predictive accuracy. All cases in each subgroup are classified either as a true or not true case. The binary partitioning process is repeated for each subgroup and the optimal decision tree with the smallest misclassification rate is selected. For this study, we developed 2 different algorithms aimed at maximizing either sensitivity or specificity. The performance of each algorithm was summarized by calculating its sensitivity (the percentage of true incident fibroid cases, as determined from medical record review, that were correctly classified as such by the algorithm), specificity (the percentage of cases determined not to be a true incident fibroid, that were correctly classified as such by the algorithm), PPV (the percentage of incident fibroid cases as classified by the algorithm that were confirmed as true cases by medical record review), and negative predictive value (the percentage of cases classified as not incident fibroid by the algorithm that were indeed not an incident case). The corresponding 95 percent CI for these measures also were calculated based on the exact binomial distribution. The CART analysis was performed using CART® 7.0 (Salford Systems, San Diego, CA).

## Results

### Accuracy of diagnosis codes for incident uterine fibroid

Among the 70 women who had no fibroid diagnosis but a potentially related procedure and/or symptom that were reviewed by a study abstractor, 42 had a heavy uterine bleeding code, 15 had hysterectomy, 10 had ablation, and 3 had both heavy uterine bleeding and ablation. None had myomectomy. Only 6 (8.6 percent) of this group were found to have a confirmed uterine fibroid, of which 4 were incident cases. The PPV for an incident fibroid was 20.0 percent from a hysterectomy code, 0 percent from an ablation code, and 2.2 percent from a heavy uterine bleeding code. Because of these very low predictive values, abstraction of these potential cases was halted early, and the development of the classification algorithms was proceeded based on the 617 potential cases selected who had a uterine fibroid diagnosis code.

The average age at the time of a uterine fibroid diagnosis among the 617 women was 47.8 years and the mean BMI was 30.4 kg/m^2^ (Table 2). The majority of these women (61.1 percent) were white. Over 80 percent had a uterine fibroid ICD-9 diagnosis code of 218.9 (fibroid, not otherwise specified). Almost half (48.6 percent) of the potential cases received their uterine fibroid diagnosis from a radiology visit, whereas 37 percent from an outpatient visit. Based on the medical record review, 583 (94.5 percent) had a confirmed fibroid, and 482 were confirmed as incident cases. PPV for a confirmed UF (including both incident and prevalent) using ICD-9 code only was 94.5 percent (95 percent CI: 92.4 percent, 96.2 percent). PPV of fibroid ICD-9 diagnosis codes for an incident fibroid was 78.1 percent (95 percent CI: 74.6 percent, 81.3 percent).

**Table 2:** Characteristics of 617 women with a uterine fibroid diagnosis code. ICD-9, International Classification of Diseases, 9^th^ revision, Clinical Modification; SD, standard deviation.

	N	%

Age at uterine fibroid diagnosis, years		
Mean (SD)	47.8	8.9
18–34	46	7.5
35–39	57	9.2
40–44	104	16.9
44–49	150	24.3
50–54	116	18.8
55–65	144	23.3
Race/Ethnicity		
Hispanic	50	8.1
White	377	61.1
African American	58	9.4
Asian/Hawaiian/Pacific Islander	93	15.1
Native American	19	3.1
Other	10	1.6
Unknown	10	1.6
Body mass index (kg/m^2^)		
Mean (SD)	30.4	7.7
<25	167	27.1
25.0–29.9	176	28.5
30.0–34.9	102	16.5
35+	158	25.6
Unknown	14	2.3
Smoking status		
Never	408	66.1
Past	153	24.8
Current	37	6
Unknown	19	3.1
Uterine fibroid ICD-9 diagnosis code*		
218 Uterine leiomyoma	0	0
218.0 Submucous leiomyoma of uterus	49	7.9
218.1 Intramural leiomyoma of uterus	71	11.5
218.2 Subserous leiomyoma of uterus	43	7
218.9 Leiomyoma of uterus, unspecified	505	81.8
Visit setting when uterine fibroid was diagnosed		
Inpatient	29	4.7
Emergency department	7	1.1
Urgent care	36	5.8
Outpatient	228	37.0
Radiology	300	48.6
Other care	17	2.8

* Diagnosis codes were not mutually exclusive.

### Incident uterine fibroid classification algorithms

**Figure 1:**
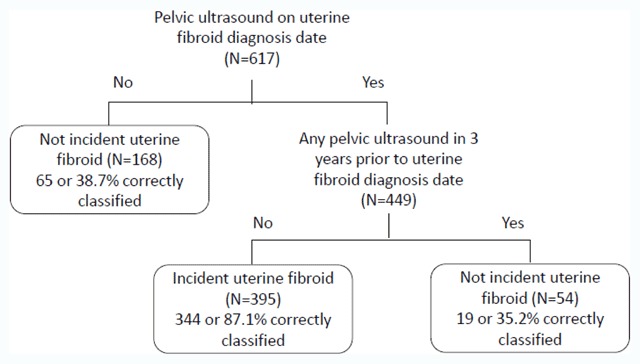
Classification algorithm with high sensitivity for identifying incident uterine fibroid cases.

Two incident uterine fibroid classification algorithms with different performance characteristics were developed. The algorithm with higher sensitivity would be useful in situations when EHR/medical record review of potential cases is available to confirm true case status (Figure 1). The strongest predictor in this algorithm was presence of a pelvic ultrasound on the fibroid diagnosis date. Of the 617 potential fibroid cases, 168 did not have a pelvic ultrasound on their diagnosis date and, by the algorithm, were classified as not an incident fibroid case (Figure 1). The remaining 449 potential cases that had a pelvic ultrasound on the diagnosis date were further classified as true incident fibroid cases if they did not have a pelvic ultrasound in the 3 years prior. Of this group of potential cases (n = 395), 344 were true incident fibroid cases. Thus, PPV for the algorithm was 87.1 percent. Sensitivity, specificity, and negative predictive value were 71.4 percent, 62.2 percent, and 37.8 percent, respectively (Figure 1).

**Figure 2:**
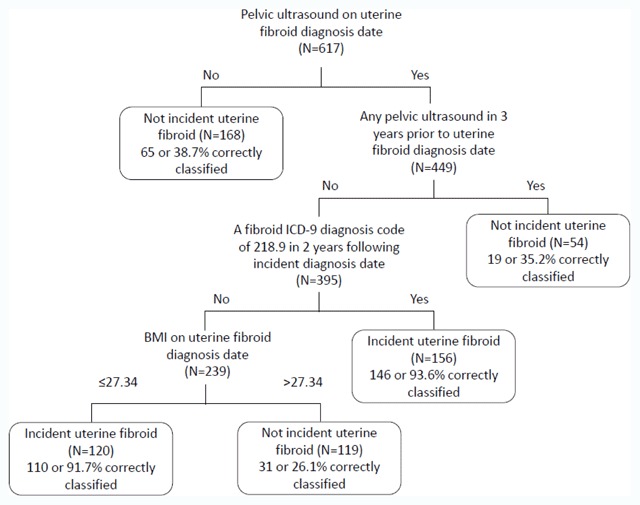
Classification algorithm with high specificity for identifying incident uterine fibroid cases. Abbreviations: BMI, body mass index; ICD-9, International Classification of Diseases, 9^th^ revision, Clinical Modification.

Figure 2 shows the algorithm with a high PPV and high specificity, which may be useful when medical record review is not feasible and, as a result, the aim is to minimize the inclusion of non-cases. This algorithm was built on the one presented in Figure 1, and further classified the 395 potential cases with a pelvic ultrasound on the diagnosis date but none in the 3 years prior as to the presence of a fibroid ICD-9 diagnosis code of 218.9 (unspecified type) in the 2 years following diagnosis and by BMI ≤ 27.3 kg/m^2^ or > 27.3 kg/m^2^. This algorithm had a higher PPV, 92.8 percent, but lower sensitivity, 53.1 percent. Specificity was 85.2 percent, and negative predictive value was 33.7 percent.

## Discussion

Our study found a good PPV (78.1 percent) of ICD-9 diagnosis codes alone for identifying incident uterine fibroids among women aged 18–65 years, when compared to a gold standard assessment from medical record review. A similar study in the UK reported a PPV of 88.8 percent for the Read uterine fibroid diagnosis codes in identifying incident fibroids among women 15–54 years of age [10,12]. In addition to assessing the accuracy of the fibroid diagnosis codes to identify incident cases, the same UK study also evaluated the Read codes for hysterectomy, uterus-preserving procedures, and heavy menstrual bleeding in the absence of a fibroid diagnosis. They found a PPV of 29.7 percent for hysterectomy codes, 57.7 percent for uterus-preserving procedure codes, and 15.9 percent for heavy menstrual bleeding codes—substantially higher than our findings of 20.0 percent, 0 percent, and 2.2 percent, respectively, although our chart review sample sizes were relatively small. These discrepancies are likely due to differences in coding systems (ICD-9 vs Read codes) and coding practices in these different health care settings. In the United States, a diagnosis code is required for all health care utilization in order to obtain reimbursement and any required payments.

Using additional potential predictors collected from the automated data, we developed 2 classification algorithms that improved upon the incident case-finding PPV of 78.1 percent that was based solely on a fibroid diagnosis code, with PPVs of 87.1 percent and 92.8 percent for algorithms I and II, respectively. The 2 strongest predictors included in both algorithms were presence of a pelvic ultrasound (based on procedure codes) on the fibroid diagnosis date and absence of pelvic ultrasound in the 3 years prior to diagnosis date. This confirms the importance of pelvic imaging in diagnosing uterine fibroid and is consistent with the literature [13]. A recent study that utilized EHR data found a 96 percent PPV for fibroids (including both incident and prevalent) from an algorithm that identified potential cases using the presence of an ICD-9 fibroid diagnosis code plus a history of pelvic imaging (ultrasound, CT, and/or MRI) [14]. In our study, PPV for a confirmed UF (including both incident and prevalent) using ICD-9 code only (regardless of presence of pelvic imaging) was 94.5 percent. Restricting to those with pelvic imaging in the 3 years prior to or on diagnosis date, PPV was similar (94.7 percent). The study by Feingold-Link et al. [14] did not distinguish incident from prevalent cases, whereas our algorithm development focused on identification of incident fibroid cases.

**Table:** 

Incident Uterine Fibroid Status		Predicted from Algorithm		Total

		No	Yes	

True Status from Medical	No	84	51	135
Record Review	Yes	138	344	482
Total		222	395	617

Algorithm Performance (95% confidence interval):

- Sensitivity = 71.4% (67.1–75.4%)
- Specificity = 62.2% (53.5–70.4%)
- Positive predictive value = 87.1% (83.4–90.2%)
- Negative predictive value = 37.8% (31.4–44.6%)

The case-finding algorithms to identify women with incident uterine fibroids can be used for research or population management. Specific research questions might help tease apart the heterogeneity of the population identified. For example, by linking specific symptoms such as bleeding, pain or infertility to specific patient phenotypes, one can more specifically test therapeutic interventions in targeted populations, either using observational methodologies or in a clinical trial. The targeting of a specific population would improve the ability of an observational study or a trial to most efficiently detect a possible beneficial intervention in a newly diagnosed patient. One could also evaluate specific characteristics such as age, socioeconomic status, smoking, or race/ethnicity among newly diagnosed patients as predictors of specific symptoms at presentation or as predictors of a treatment response.

The 2 case-finding algorithms that we developed are simple and can be readily implement using widely available EHR data. They can provide different emphases for case selection, based on the needs and resources of the study. The first algorithm only relying on the presence of pelvic ultrasound would be useful if the aim of the study were to identify and include as many true cases as possible and medical record review was feasible to reclassify false positive cases (Figure 1). On the other hand, if medical record review was not feasible and the objective of the study was to obtain a more pure (and thus, smaller) group of true incident cases by relying on electronic data alone, then the second algorithm that also incorporated specific fibroid code information and BMI could be used (Figure 2). This algorithm included capture of women who did not have pelvic imaging studies but were slender enough (BMI ≤ 27.3 kg/m^2^) that most likely providers felt relatively confident assigning an ICD-9 diagnostic code of uterine fibroid based on the history and pelvic examination alone. Within our health care organization, if a diagnosis can be made with relative certainty without additional procedures that will not change management decisions, this practice is encouraged and diminishes unnecessary health care costs.

**Table:** 

Incident Uterine Fibroid Status		Predicted from Algorithm		Total

		No	Yes	

True Status from Medical	No	115	20	135
Record Review	Yes	226	256	482
Total		341	276	617

Algorithm Performance (95% confidence interval):

- Sensitivity = 53.1% (48.5–57.6%)
- Specificity = 85.2% (78.1–90.7%)
- Positive predictive value = 92.8% (89.0–95.5%)
- Negative predictive value = 33.7% (28.7–39.0%)

These case-finding algorithms were developed using CART that has several advantages. First, CART is a nonparametric approach that makes no distributional assumptions regarding the potential predictors and their relationship to the outcome. Second, it can handle a large number of potential predictors and efficiently evaluates all possible binary splits and interactions between all of them. Finally, the resulting algorithms are visually displayed, and are easy and straightforward to interpret. An additional strength of this study was our ability to review cases with other possibly related procedures or conditions (hysterectomy, uterus-preserving procedure, and abnormal uterine bleeding) in the absence of a fibroid diagnosis code to confirm that we did not miss many true cases that did not have a fibroid code. We utilized data from the VDW, which is a common data model for the health care plans within the HCSRN. Our algorithms are simple, mostly utilizing procedure and diagnosis codes that can be easily applied to other sites with the VDW or to sites with comprehensive capture of utilization data that are now widely available from many electronic databases. Thus, these algorithms potentially can be applied more broadly to other health care settings.

A limitation of our study was that the analyses were conducted in a single health plan where routine pelvic ultrasound was not done, and thus, results may not be widely generalizable. However, our algorithms identified the presence of prior pelvic ultrasound as the most important predictor of an incident fibroid. Together with ICD-9 diagnosis code, we observed a very similar PPV for fibroids as in a recent study that also utilized similar EHR systems [14]. It was beyond the scope of our study to validate these algorithms’ performance by applying them to data from another health plan.

## Conclusion

To our knowledge, this is the first study to develop algorithms using EHR data to refine case-finding for incident uterine fibroids. We found a good PPV of the ICD-9 fibroid diagnosis codes to identify incident cases from electronic data sources, which adds to the limited information in the available literature. We also offer a strategy to more accurately identify incident uterine fibroid cases through the use of additional electronic data. The algorithms improved upon the diagnosis codes alone both by improving PPV and tailoring for higher sensitivity or specificity. Future studies seeking to identify incident fibroid cases from electronic data in order to evaluate new or existing medical therapies for uterine fibroids may find these strategies useful.
